# Supplementary material for: Diverse cell junctions with unique molecular composition in tissues of a sponge (Porifera)
Source: EvoDevo. 2019 Oct 29;10:26. doi: 10.1186/s13227-019-0139-0 (PMC6820919; doi:10.1186/s13227-019-0139-0)
Supplement: Supplementary file 4 — Additional file 4. Supplementary Figures [file 13227_2019_139_MOESM4_ESM.pdf]

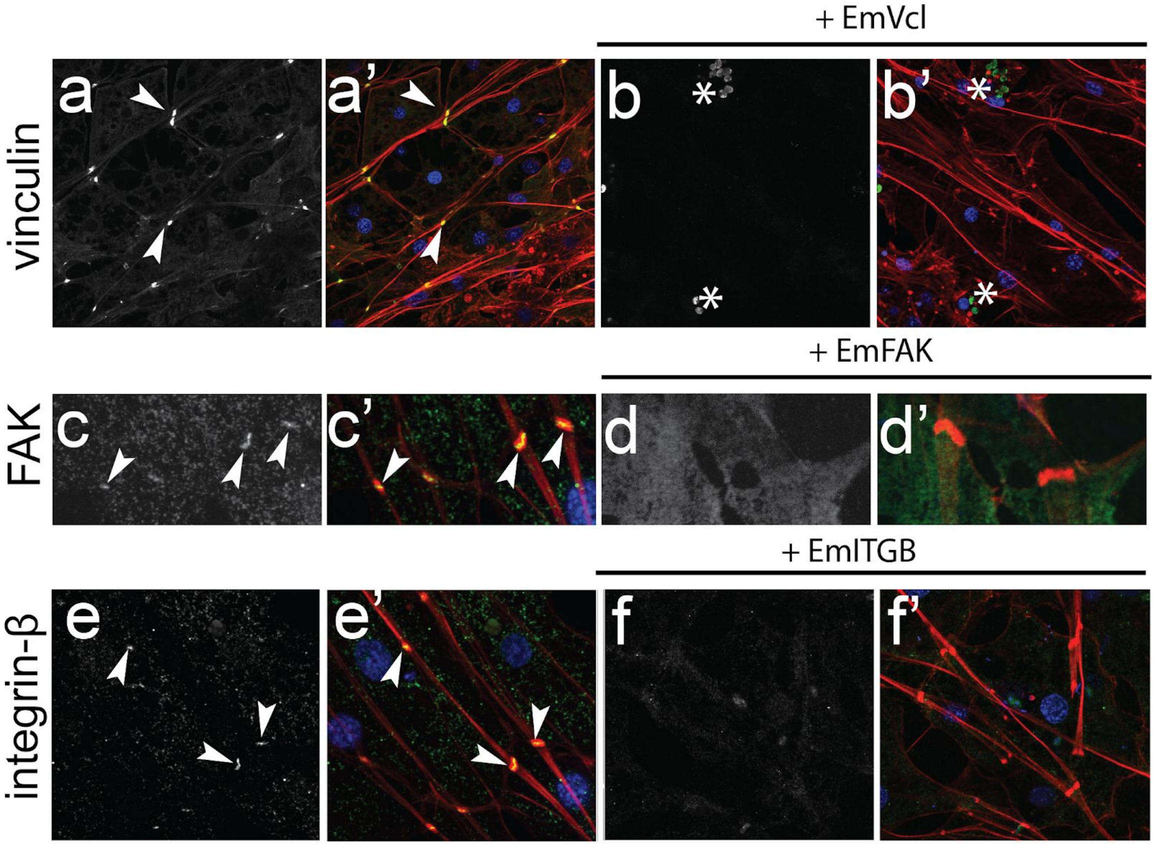

Supplemental Figure 1: Antibody validation by immunostaining preadsorption assay. Immunostained cell-cell junctions in the apical endopinacoderm using control (left panels) versus pre-adsorbed antibody aliquots. Preadsorption with 1-10 μg of each recombinant antigen completely abolished observed staining patterns, supporting the specificity of the immunostaining signal. [a-f = antibody only; a'-f' = F-actin (red), DNA (blue) antibody (green)].

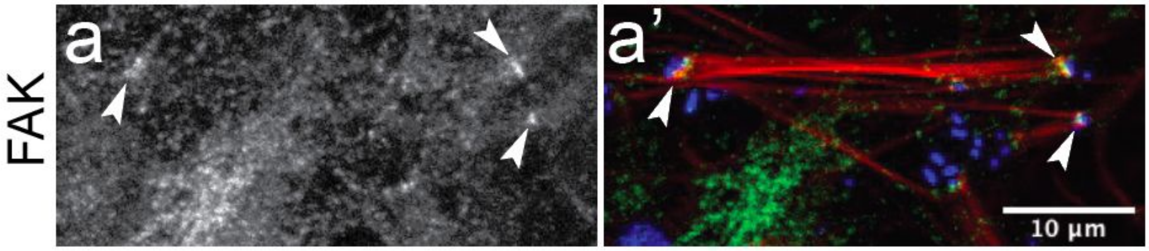

Supplemental Figure 2: EmFAK was rarely detected at Bacteria-Associated Stress Fibers Whereas EmFAK was not routinely detected at focal-adhesion like structures in the basopinacoderm, it was sometimes present at the distal ends bacteria-associated stress fibers. It is unclear if this reflects a dynamic process or something about the maturation state of these junctions, or if the epitope recognized by anti-EmFAK is simply less accessible at these structures. [a = antibody only; a’ = F-actin (red), DNA (blue) EmFAK (green)].

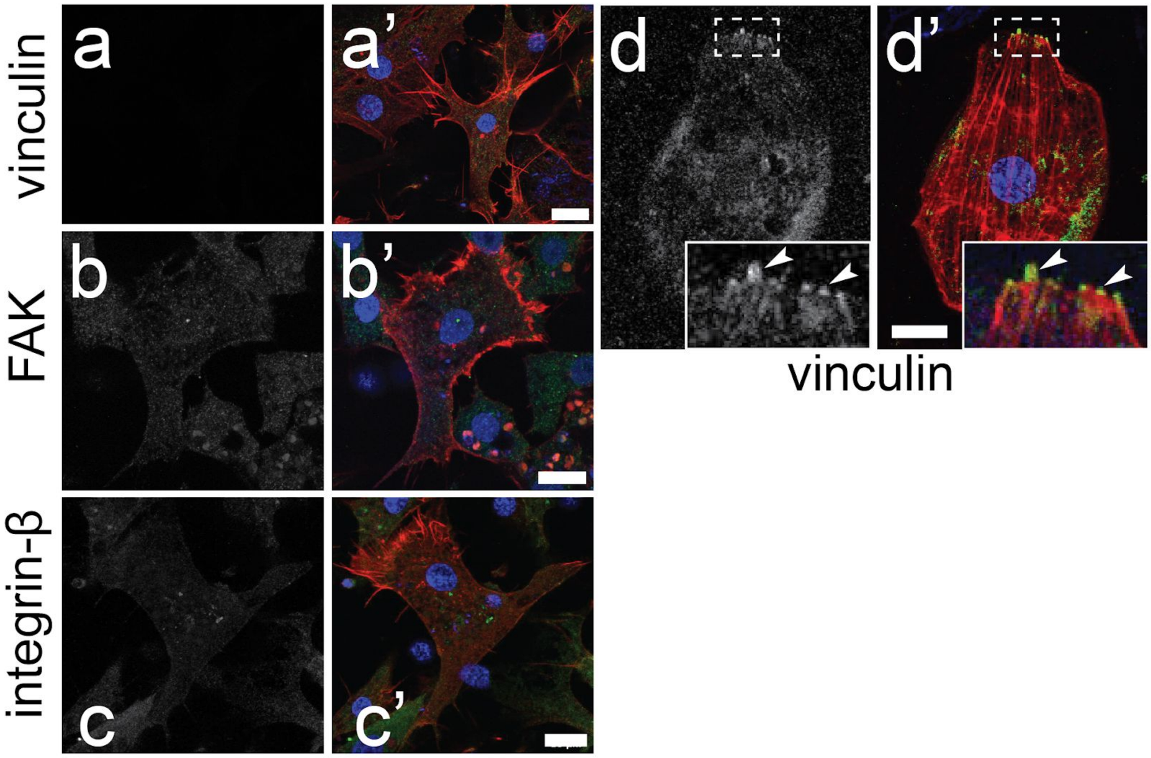

Supplemental Figure 3: Migratory cells form focal adhesion like structures in two-dimensional, but not three-dimensional environments

Migratory cells in the mesohyl where stained for (a) EmVcl, (b) EmFAK and (c) EmITGB. Focal adhesion-like structures were not detected in this three-dimensional environment. (d) Basopinacocytes sometimes detach from the leading edge of the spreading juvenile sponge and migrate on the coverslip. In this context, EmVcl was detected at focal adhesion-like structures. Arrowheads indicate EmVcl positive focal adhesion-like structures. [(a-d) antibody staining only; (a’-d’) antibody = green, DNA = blue, F-actin = red; scale bar = 10μm].

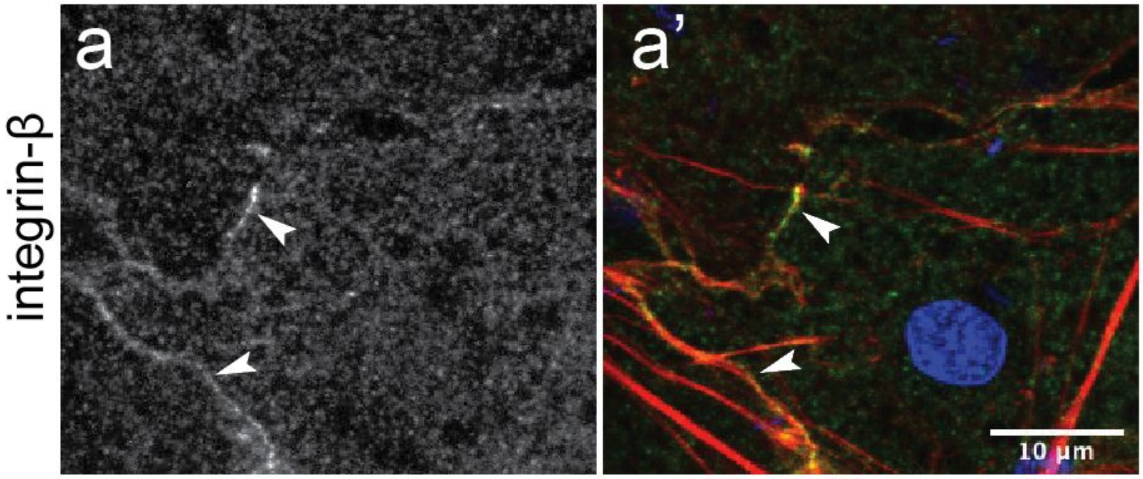

Supplemental Figure 4: EmITGB detected at cell boundaries in the basopinacoderm
EmITGB staining was patchy and of low intensity at cell boundaries in the basopinacoderm of early juveniles. [a = antibody only; a' = F-actin (red), DNA (blue), EmITGB (green].

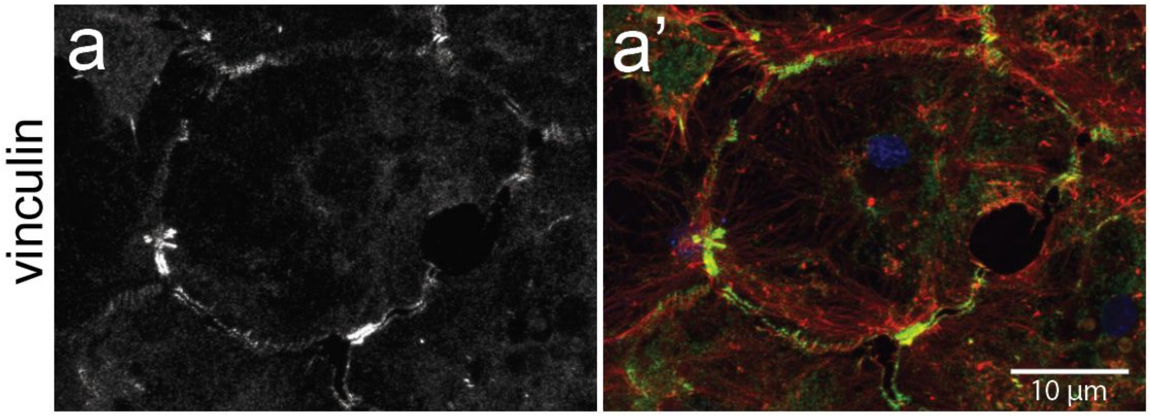

Supplemental Figure 5: Robust cell-cell junctions in the basopinacoderm of 1 month old sponges.
EmVcl was patchy and of low intensity in early juveniles, but robust in older (1 month) tissues, which formed cell-cell junctions similar to those in the apical endopinacoderm. [a = antibody only; a’ = F-actin (red), DNA (blue), EmVcl (green)].

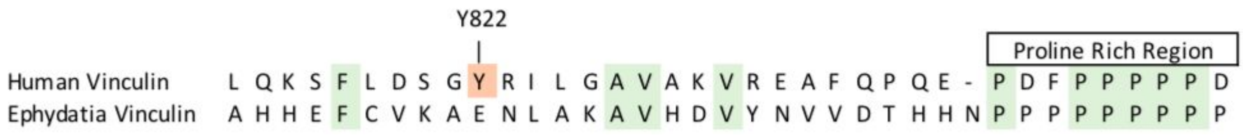

Supplemental Figure 6: Alignment of Y822 region of *E. muelleri* and human vinculin
Tyrosine 822 (Y822) of human vinculin is phosphorylated by Abelson kinase upon recruitment to the adherens junction. The homologous region of EmVcl (just before the proline rich region) contains a tyrosine residue but has low sequence conservation with human vinculin.

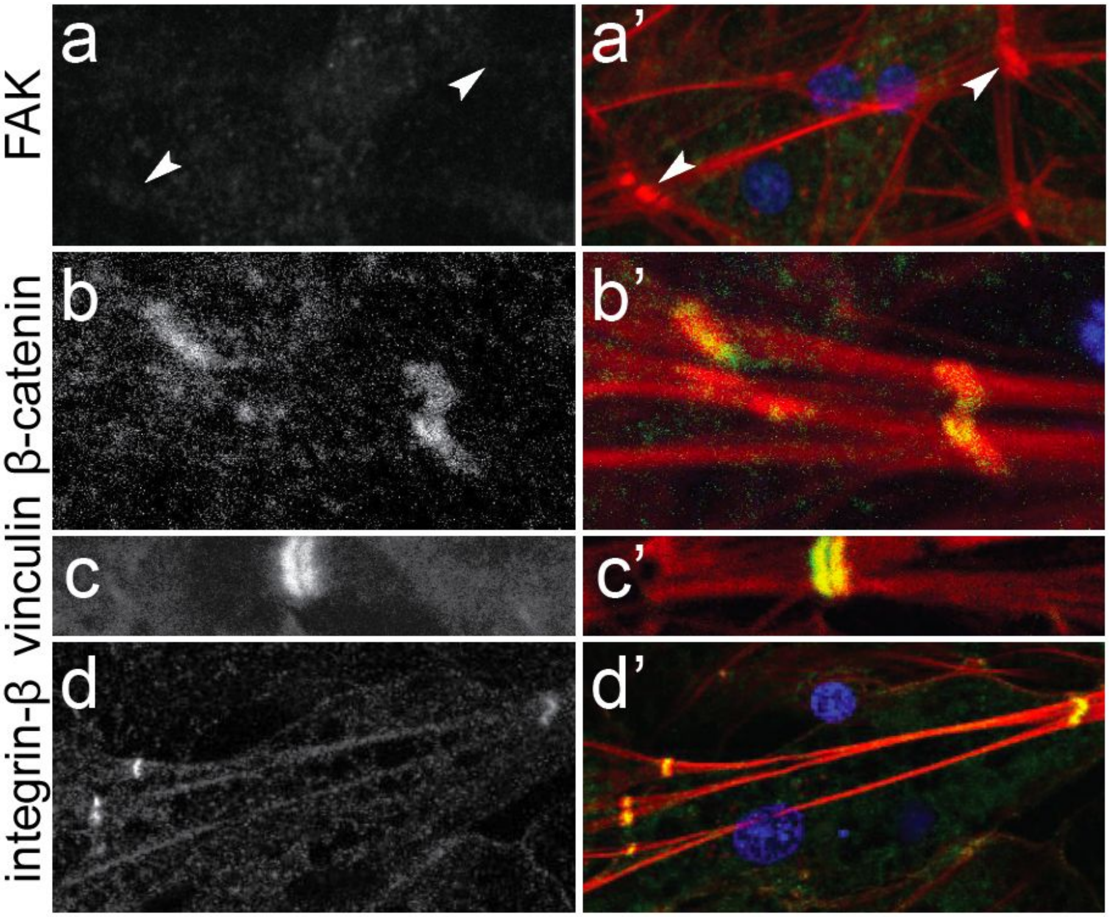

Supplemental Figure 7: FAK inhibitor 14 effects on cell-cell junction composition
(a) Treatment with 5 μM FAK inhibitor 14 (FAKi 14) abolished FAK staining at cell-cell junctions in the apical endopinacoderm, but had no effect on (b) Emβ-catenin, (c) EmVcl or (d) EmITG to staining. [a-d = antibody only; a’-d’ = F-actin (red), DNA (blue), antibody (green)].

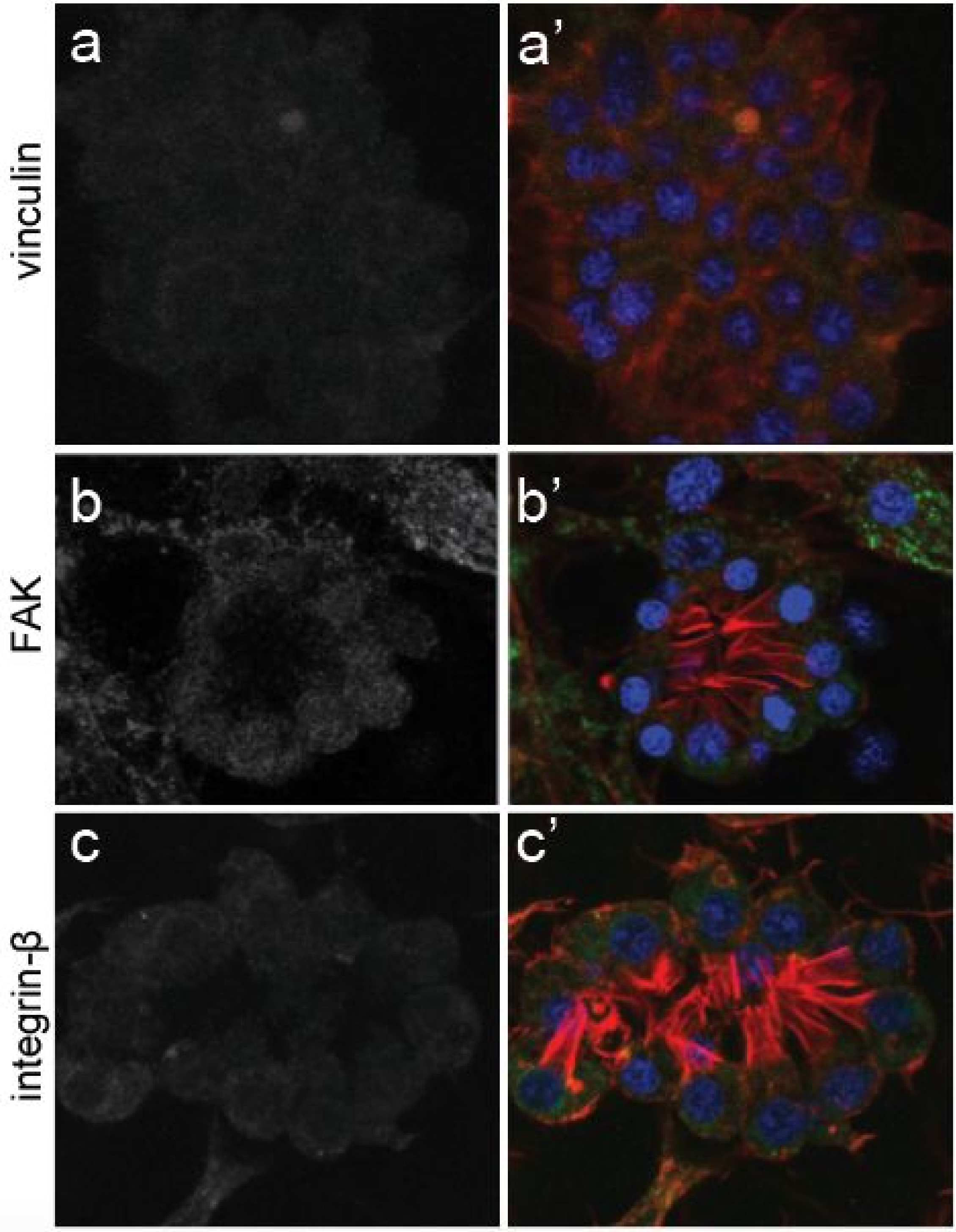

Supplemental Figure 8: Focal adhesion proteins are not detected in the choanoderm
No staining was detected for focal adhesion proteins in the choanoderm. [a-c = antibody only; a’-c’ = F-actin (red), DNA (blue), antibody (green)].
